# The Western Dental Journal’s Criticism

**Published:** 1888-07

**Authors:** 


OUR esteemed contemporary, *The Western Dental Journal*, in commenting on the announcement in the *Ohio Journal of Dental Science* of the establishment of a three years' graded course in the University of Michigan, lays itself open to being misunderstood. *Ye* Editor surely does not desire to place himself in opposition to higher education or in the position of wishing to limit the possibilities of dental education. He says:—

"If the University of Michigan wishes to teach preliminary science in connection with dentistry, well and good; let it increase the length of its term as much as it will; but if it expects to continue as a purely dental school, its professors cannot possibly find material to fill their time except by padding their lectures with extraneous matter more or less foreign to the subject of dentistry."

In the interest of the profession we would ask the doctor, what is pure dentistry? There exist "vast possibilities" for differences of opinion on this question. We most fully believe that a dentist should be educated with the view of practicing dentistry—that he should be a dentist first, last and all the time; but that he can know too much of the collateral branches we most strenuously deny. Dentistry is a branch of the healing art, and those who practice it as such should be as broadly educated as their means, time and capacity will admit. We most heartily coincide with the stand taken by the University of Michigan, and hope to see many of our schools fall in line. Those schools that do not will necessarily have to accept a back seat, and voluntarily relegate themselves to the position of inferior schools. No college can offer as full instruction in two terms of five months each as can schools that require three terms of nine months each, no matter how good their facilities. The capacity of students to absorb is limited, and no amount of "cramming" will ever stand instead of time given to actual practical work. A student may "book up" for an examination, but not for a life-work.

---

ANNOUNCEMENT.

